# A comparative analysis of methods for *de novo* assembly of hymenopteran genomes using either haploid or diploid samples

**DOI:** 10.1038/s41598-019-42795-6

**Published:** 2019-04-24

**Authors:** Tal Yahav, Eyal Privman

**Affiliations:** Department of Evolutionary and Environmental Biology, Institute of Evolution, University of Haifa, Haifa, 3498838 Israel

**Keywords:** Genomics, Genome informatics

## Abstract

Diverse invertebrate taxa including all 200,000 species of Hymenoptera (ants, bees, wasps and sawflies) have a *haplodiploid* sex determination system, where females are diploid and males are haploid. Thus, hymenopteran genome projects can make use of DNA from a single haploid male sample, which is assumed advantageous for genome assembly. For the purpose of gene annotation, transcriptome sequencing is usually conducted using RNA from a pool of individuals. We conducted a comparative analysis of genome and transcriptome assembly and annotation methods, using genetic sources of different ploidy: (1) DNA from a haploid male or a diploid female (2) RNA from the same haploid male or a pool of individuals. We predicted that the use of a haploid male as opposed to a diploid female will simplify the genome assembly and gene annotation thanks to the lack of heterozygosity. Using DNA and RNA from the same haploid individual is expected to provide better confidence in transcript-to-genome alignment, and improve the annotation of gene structure in terms of the exon/intron boundaries. The haploid genome assemblies proved to be more contiguous, with both contig and scaffold N50 size at least threefold greater than their diploid counterparts. Completeness evaluation showed mixed results. The SOAPdenovo2 diploid assembly was missing more genes than the haploid assembly. The SPAdes diploid assembly had more complete genes, but a higher level of duplicates, and a greatly overestimated genome size. When aligning the two transcriptomes against the male genome, the male transcriptome gave 2–3% more complete transcripts than the pool transcriptome for genes with comparable expression levels in both transcriptomes. However, this advantage disappears in the final results of the gene annotation pipeline that incorporates evidence from homologous proteins. The RNA pool is still required to obtain the full transcriptome with genes that are expressed in other life stages and castes. In conclusion, the use of a haploid source material for a *de novo* genome project provides a substantial advantage to the quality of the genome draft and the use of RNA from the same haploid individual for transcriptome to genome alignment provides a minor advantage for genes that are expressed in the adult male.

## Introduction

Whole genome *de novo* assembly is a crucial component in various types of genetic research. It is the foundation for the development of genetic resources such as gene annotation, high resolution maps of polymorphism, genomic structural variation, etc. These resources enable a wide range of applications involving genomic, transcriptomic, or epigenomic analysis in fields including biomedicine, agriculture, biotechnology, molecular ecology, and evolutionary biology^1^. Ideally, a fully sequenced genome, with long contiguous genomic segments anchored to full-length chromosomes should be produced, often by combining sequencing and mapping technologies. Such a genome project demands substantial funding, which is often reserved for medical or agricultural research. However, the rapid development of Next Generation Sequencing (NGS) over the last decade provided a relatively affordable and powerful tool fitting also for non-model organism research, where lower-quality draft genome assemblies are typically produced. Two main challenges affect *de novo* assembly of eukaryotic genomes: (1) repetitive sequences, including gene duplications, transposons, and short sequence repeats; and (2) polymorphism, including single-nucleotide polymorphisms (SNPs), insertions and deletions, and large genome rearrangement polymorphisms. Although sequencing technologies have advanced dramatically in the past decade, these issues still present a major hurdle, resulting in highly fragmented assemblies.

The aforementioned advancements led to an exponential growth of genomic data produced with short reads sequencing technologies and invoked the need for more capable assemblers, incorporating new computational approaches. Many of the short-read assemblers use a *de-Bruijn* graph representation in the process of genome assembly. The graph connects short sequence fragments based on their overlapping subsequences (*k*-mers). A contiguous genomic sequence (*contig*) is assembled based on a path through the graph. During the *de-Bruijn* graph walkthrough, the assembler must deal with repetitive elements by resolving alternative or circular paths (“bubbles”). However, this is often impossible when extending contigs through repetitive sequences longer than the read length^2^ and typically results in a highly fragmented assembly, consisting of non-repetitive fragments ending in unresolved repetitive sequences^3^. Long-insert sequencing protocols (e.g., the mate-pair protocol) can be employed to spatially associate and order contigs to create larger fragments (*scaffolds*), thereby overcoming repetitive elements shorter than the insert size^3^.

Genetic variations across the genome, such as SNPs, are another challenge for the assembler to tackle. The level of polymorphism or heterozygosity vary considerably among species, and highly polymorphic species such as amphioxus are more challenging^4^. The assembler attempts to recognize heterozygous sites in the genome, and collapse them so that only one allele is present in the resulting reference sequence. However, the assembler might also collapse sequences that are in fact slightly different variants of a repetitive sequence. Conversely, it might treat multiple alleles as a duplicated versions of a repetitive sequence and include them in the assembly^5^. Higher ploidy levels introduce an even greater challenge relative to the ‘commonplace’ diploid organisms. Genome projects of polyploid organisms such as the tetraploid African clawed frog (*Xenopus Laevis*)^6^ or the hexaploid bread wheat (*Triticum aestivum*)^7^ are considerably more complex than diploid genomes. Conversely, assembling a genome from a haploid source is not affected by polymorphism, and is expected to facilitate higher contiguity in the genome assembly^5^. The availability of haploid samples may benefit genome projects for species with haplodiploid sex determination – a mechanism found in many invertebrates, where females develop from fertilized eggs and males from unfertilized eggs. The largest haplodiploid animal clade is the Hymenoptera, including more than 200,000 species of ants, bees, wasps, and sawflies. This approach was already put into practice in previous hymenopteran genome projects, such as the leafcutter ant *Acromyrmex echinatior*^8^ and the fire ant *Solenopsis invicta*^9^, which used haploid males as their main source for genome sequencing and assembly, alongside a pool of workers for transcriptome sequencing.

The goal of the presented study was to quantify the advantage in sequencing a haploid male as opposed to a diploid female sample, using the ant species *Cataglyphis niger*. Furthermore, the advantage of using both RNA and DNA from the same male individual was evaluated, with the expectation that this will provide greater confidence in transcript-to-genome alignment, and improve the annotation of gene structures in terms of their exon/intron boundaries.

## Materials and Methods

### Samples

All samples were collected from *Bezet* beach in northern Israel. This population was previously described as *C. drusus*^10^, but our recent species delimitation study showed that *C. drusus* is not a separate species from *C. niger*, because these populations are not differentiated by their nuclear genomic DNA^11^. Male and worker samples were collected from the same nest while samples for the RNA pool were collected from several additional nests in the same site (33°4′40.88″N/35°6′33.97″E). Samples were brought to the lab and snap-frozen in liquid nitrogen. Both DNA and RNA were extracted from one haploid male using the All-Prep DNA & RNA mini extraction kit (QIAGEN). Diploid males are occasionally produced in some haploidiploid species, so it was important to verify the haploidy of the sample used for genome sequencing. We used four highly polymorphic microsatellite loci verify that the male sample has a single allele in each locus (Supplementary Table S5) as previously described^12^. The lack of heterozygosity in four highly polymorphic microsatellites strongly indicates that the sample was haploid, because a diploid sample has an expected probability of 2.76% to have homozygous genotypes in all four loci, based on the heterozygosity level of each of these loci in the *Bezet* population of *C. niger* (Tali Reiner-Brodetzki, personal communication). Additionally, the diploid DNA sample was extracted using the DNeasy Blood & Tissue kit (QIAGEN), which is identical to the DNA extraction in the All-Prep protocol.

An RNA pool sample was obtained from whole-body RNA extracts from several individuals from three different nests collected in the *Bezet* site: one worker, one gyne, one male, five larvae of three different size groups and three pupae from two different size groups. The diversity in the RNA pool composition should provide a comprehensive representation of the transcriptome of *C. niger*, including genes and alternative splice isoforms expressed only in certain life stages and/or castes. Sample extraction was performed separately for each sample type, using the RNeasy mini kit (QIAGEN). RNA concentration was measured using *NanoDrop* UV spectrophotometer (ND2000; Thermo-Fisher Scientific) and the RNA quantity was normalized to achieve equal representation of each sample type (caste/developmental stage) in the final pool.

### Sequencing

DNA and RNA sequencing was performed using the HiSeq 2500 sequencing platform (Illumina) and the HiSeq SBS Kit v4 chemistry (Illumina) by *Eurofins Genomics GmbH* (Germany). Genomic DNA libraries were prepared using NEBNext Ultra DNA Lib Prep Kit for Illumina (E7370). For genomic DNA sequencing, two paired-end libraries were constructed for each sample (haploid/diploid) with insert sizes of 300 and 550 bp. Each pair of libraries was multiplexed in one lane, giving a total coverage for the 300 bp library of 84X/127X for the haploid/diploid samples respectively, and 93X/108X for the 550 bp library. Coverage is calculated considering a genome size estimation of 220Mbp, based on flow cytometry measurements according to the methodology described in Aron *et al*.^13^ (Hugo Darras, personal communication). Following poly-A enrichment for mRNA sequencing, a paired-end, strand-specific cDNA library was constructed for each sample (male and pool) using NEBNext Ultra Directional Lib Prep Kit for Illumina (E7420). Each RNAseq library was sequenced in a separate lane. Average insert sizes of each library were estimated using a TapeStation instrument (Agilent).

Quality control of the raw sequence data was performed using FastQC (version 0.11.5)^14^. Trimmomatic (version 0.32)^15^ was used to remove or trim low quality reads and adaptor contamination. Reads trimmed to less than 80 bp were removed. A sliding window of four bases was applied and ends of reads were trimmed where the window-averaged quality score (Phred score) was lower than 15. Reads were randomly removed from the male genomic libraries and from the pool transcriptome library to equalize samples for a fair comparison (Table 1). Both DNA and RNA %Q30 scores were above 88% (percentage of bases with a quality score of at least 30, which indicates base call accuracy of 99.9%).

**Table 1:** Illumina libraries constructed. Two genomic DNA libraries (300, 550 bp) constructed from each of the source materials. For RNA sequencing, one library for each source was constructed.

DNA							
Sample type	Library type	Average insert size [bp]	No. of reads raw data [bp]	%Q30^b^	Mean Q^a^	No. of reads after trimming & reduction [bp]	Final depth [X]
Male (Haploid)	Paired-end 125b × 2	290	84,756,564	90.34	34.28	74,249,558	84.4
Male (Haploid)	Paired-end 125b × 2	510	95,513,084	88.63	33.9	82,214,665	93.4
Worker (Diploid)	Paired-end 125b × 2	340	120,165,717	93.15	35.21	74,661,870	84.8
Worker (Diploid)	Paired-end 125b × 2	560	105,253,786	90.44	34.56	83,032,369	94.4
**RNA**							
Male	Paired-end 125b × 2	210	184,860,252	93.07	35.13	173,306,486	N/A
Pool	Paired-end 125b × 2	240	173,787,824	93.5	35.22	173,787,824	N/A

^a^Mean Phred Q scores for each of the libraries, ^b^%Q30 is the percentage of bases with a quality score of at least 30 for each library.

### Genome assembly

In this study, we used two popular assemblers: SOAPdenovo2 (version r240)^16^ and SPAdes (version 3.9.1)^17^ to assemble the genome from either the haploid or diploid samples. Although both all three assemblers rely on a *de-Bruijn* graph, they differ in their use of this method to overcome challenges in the assembly process. SOAPdenovo2 takes a more standard approach by using a single, user-defined *k*-mer for the contiging process^16^, while SPAdes was inspired by a theoretical approach termed “paired *de-Bruijn* graphs” (PDBG)^18^. SPAdes implements a *k*-bimer adjustment method, which utilizes read-pairs to create a paired *de-Bruijn* graph. SPAdes assembles the genome using multiple *k*-mer sizes and eventually combines them into one consensus sequence. It was originally designed for prokaryotic genomes but was later developed to accommodate large eukaryotic genomes^17^. The two assemblers were configured with default parameter settings regarding error correction cutoffs, etc. With SOAPdenovo2, several assemblies were constructed using different *k*-mer sizes (35, 45, 63, 85, and 115). Among those, the 115 *k*-mer size was chosen being the one with the highest contig and scaffold N50 sizes (defined as the minimal contig/scaffold length such that at least half of the total assembly length will be found in contigs/scaffolds of that size or bigger; calculated using the PERL script https://gist.github.com/standage/5526823). SPAdes was run using an array of *k*-mer sizes ranging from 13 to 123 bp.

There are multiple methods of assessing the quality of a genome assembly, yet no single one of them is considered a sufficient on its own for assembly quality comparison^19^. Therefore, we evaluated the alternative genome assemblies using three methods: (1) the N50 sizes of contigs and scaffolds; (2) genome completeness assessed using BUSCO (version 1.22)^20^ against the Eukaryota OrthoDB gene dataset (version 9.1; *odb9;* http://www.orthodb.org/) and Arthropoda dataset (http://busco.ezlab.org/); and (3) misassemblies detected using QUAST (version 4.4)^19^ by comparison to the genome assembly of *C. hyspanica* (Hugo Darras, unpublished data).We quantified the heterozygosity of the diploid genome by mapping the worker’s sequencing reads (two libraries 300 and 550 bp) against the male genome assembly and calling variants using GATK^21^ (version 3.7). We excluded reads that were mapped to multiple loci and filtered variants that had a minimal sequencing depth of 45X.

### RNA mapping and transcriptome assembly

Intuitively, mapping RNA sequences to a genome assembled from the same individual’s DNA should produce better annotation results. To test this hypothesis, we used BUSCO to calculate the completeness of transcripts annotated based on either the male or pool RNA, mapped against the SPAdes male genome assembly. The same BUSCO evaluation was done for the male and pool RNA against the SPAdes worker genome assembly (Supplementary Table S6). In addition, we filtered the pool RNA and left only reads which were perfectly aligned to the male genome (reads with alignment score equal to zero (AS = 0) and no base mismatch (XM = 0)) and assembled them. Mapping was done using the Tuxedo suite pipeline^22^. Bowtie2 (version 2.3.2)^23^ and Tophat2 (version 2.1.1)^24,25^ were used for indexing and mapping of RNA reads to the genome. Transcript assembly was done using Cufflinks (version 2.2.1)^26^. For the completeness evaluation we restricted BUSCO’s Arthropoda dataset to genes with up to a twofold difference in expression level between the male and the pool transcriptomes, based on normalized counts (Fragments Per Kilobase Million (FPKM) reported by Cufflinks; 476 genes overall). Basic Local Alignment Search Tool (BLAST; *tbalastn* as part of the BUSCO pipeline) annotation results were evaluated manually by comparing the alignment of the RNA reads, to the haploid genome by Tophat2 and the resulting exon and transcript annotation by Cufflinks. Visualization was done using the Integrative Genomic Viewer (IGV; version 2.3.97)^27^.

### Genome annotation

Transcripts annotation for all four genome assemblies was performed using the MAKER annotation pipeline (version 2.31.9)^28^. MAKER predicts gene structure by RNA-to-genome alignments (by Cufflinks), translated alignment of amino acid sequences of homologous proteins of related species (by BLAST/Exonerate; Supplementary Table S4), and *ab initio* gene prediction (by Augustus using the *Apis mellifera* pre-trained model). Completeness evaluation of the annotated gene set was done using BUSCO against the same restricted Arthropoda dataset, as done on the Cufflinks transcripts prior to MAKER (476 genes).

## Results

### Quality of genome assemblies

The haploid and diploid samples were each assembled using two assemblers: SOAPdenovo2 with a *k*-mer size of 115 bp, and SPAdes with *k*-mer size ranging between 13–123 bp. The four resulting assemblies were evaluated and compared. N50 contig and scaffold sizes were at least threefold larger for the haploid relative to the diploid assemblies, both by SOAPdenovo2 and SPAdes (Table 2). The SPAdes haploid assembly had almost five fold larger contig N50 size than its SOAPdenovo2 counterpart did, while the same comparison on the diploid assemblies shows a factor of ten. Scaffold N50 sizes was similar for both assemblers for the haploid sample, whereas the diploid scaffold N50 size was more than threefold higher in SPAdes than in SOAPdenovo2. The total size of the two haploid assemblies (219 Mb for SPAdes; 296 Mb for SOAPdenovo2) was much closer to the expected value of 220 Mb based on the flow cytometry measurements in *C. hyspanica*. The diploid SPAdes assembly was clearly greatly inflated – at 759 Mb. The diploid assembly size by SOAPdenovo2 (345 Mb) was not as high, yet it was still substantially higher than the expected size. Completeness evaluation by BUSCO gave mixed results (Table 2). While the haploid assembly by SOAPdenovo2 achieved better completeness than the diploid (63% vs. 47% for the Arthropoda dataset), the SPAdes assembly completeness was better for the diploid assembly (84% vs. 68%). However, the percentage of duplicated genes in the SPAdes diploid assembly was ten times higher than its haploid counterpart (38% vs. 3.9%). A similar trend can be seen in BUSCO results against the Eukaryota dataset. Misassembly detection by QAUST revealed approximately twice more misassemblies in the SPAdes haploid assembly relative to the diploid one (Table 2). An opposite trend was seen in the SOAPdenovo2 assemblies. Relative to SPAdes, both haploid and diploid SOAPdenovo2 assemblies had a dramatically higher number of local misassemblies.

**Table 2:** N50 contig and scaffold sizes for the different genome assemblies.

N50								
	SPAdes				SOAPdenovo2			
	Male (Haploid)		Worker (Diploid)		Male (Haploid)		Worker (Diploid)	
contigs	15,206		5,367		3,143		554	
scaffolds	17,901		5,742		16,307		1,659	
Total assembly size	296 Mb		759 Mb		219 Mb		345 Mb	
**Misassemblies**								
	**Male (Haploid)**		**Worker (Diploid)**		**Male (Haploid)**		**Worker (Diploid)**	
	**contigs**	**Scaff**.	**contigs**	**Scaff**.	**contigs**	**Scaff**.	**contigs**	**Scaff**.
Global	420	438	231	272	482	504	698	546
Local	465	496	259	301	2964	766	8047	753
**Completeness**								
**(a) Arthropoda**	**SPAdes**				**SOAPdenovo2**			
	**Male (Haploid)**		**Worker (Diploid)**		**Male (Haploid)**		**Worker (Diploid)**	
Complete	1832	68.0%	2256	84.0%	1706	63.0%	1276	47.0%
*Single copy*	1726	64.1%	1222	46.0%	1643	60.7%	1179	43.4%
*Duplicated*	106	3.9%	1034	38.0%	63	2.3%	97	3.6%
Fragmented	771	28.0%	376	14.0%	860	32.0%	1054	39.0%
Missing	72	2.6%	43	1.6%	109	4.0%	345	12.0%
2675 total BUSCO genes								
**(b) Eukaryota**	**Male (Haploid)**		**Worker (Diploid)**		**Male (Haploid)**		**Worker (Diploid)**	
Complete	279	92.0%	296	97.0%	260	85.0%	223	73.0%
*Single copy*	244	81.0%	59	19.0%	248	81.1%	183	60.0%
*Duplicated*	35	11.0%	237	78.0%	12	3.9%	40	13.0%
Fragmented	19	6.2%	2	0.6%	32	10.0%	63	20.0%
Missing	5	1.6%	5	1.6%	11	3.6%	17	5.6%
303 total BUSCO genes								

Misassemblies are classified as local if flanking sequences on both sides are gaped or overlapped by >85 bp and <1Kb. Global misassemblies are >1Kb.

**Figure 1:**
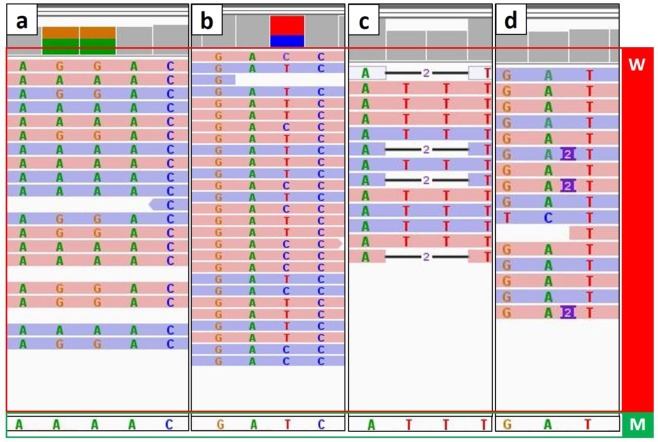
Example of SNPs and indels in the worker (diploid) DNA sequence reads mapped against the male (haploid) genome assembly. Each colored row is a read from the diploid sample (red rectangle labeled W). The sequence of the male assembly is shown at the bottom of the figure (green rectangle labeled M). Examples are shown for base substitutions (**a**,**b**), deletion (**c**), and insertion (**d**).

Variant calling of the diploid sample shows 0.11% heterozygosity of SNP (334,605 sites) and 0.02% heterozygosity of indels (46,709 sites). Figure 1 shows a few examples of SNPs and indels in the diploid worker DNA aligned against the SPAdes assembly of the male genome.

### Mapping of transcriptomes to the haploid genome

Gene structure annotation by Cufflinks was based on RNAseq reads from either the male or the pool samples, mapped against the SPAdes haploid assembly (MvM and PvM). The pool transcriptome included more expressed loci than the male transcriptome (59,386 in pool vs. 53,994 in male). This was expected because many genes are known to be specific to certain developmental stages or to specific castes. To assess the accuracy of gene models for those genes that are equally represented in both transcriptomes, we restricted the comparison to genes with no more than twofold difference in expression level between the male and pool samples. Completeness evaluation of Cufflinks transcripts (before MAKER annotation) based on the male RNA mapped to the male genome (MvM) resulted in a higher count of complete genes than pool RNA mapped to the male genome (PvM): 308 vs. 300 genes, out of a total of 476 (Table 3). We then used the MAKER pipeline to combine the Cufflinks results with evidence from protein homology and *ab initio* gene prediction, which is commonly done to obtain the most complete gene annotations. After MAKER, the number of complete genes in PvM was slightly higher than MvM: 384 vs. 382. BUSCO results for the Cufflinks transcripts found 8.8% and 9.9% duplicated genes for PvM and MvM, respectively, but these duplicates disappeared after MAKER. Fragmented gene count was higher by three genes in the PvM Cufflinks transcripts, relative to MvM. The missing gene count between the two Cufflinks transcriptomes was higher by five genes for PvM. Post MAKER annotation, missing gene counts were the same for both MvM and PvM. MAKER succeeded in correcting some of the fragmented genes, and reduced their number from 99 to 46 and from 102 to 44, in MvM and PvM respectively (Table 3). We also attempted to filter from the pool sample only reads that were 100% identical to the genome. This would result in subset that is equivalent to the male’s own RNAseq data. The Cufflinks and MAKER gene models based on this subset were intermediate between the PvM and MvM results, with 304 and 382 complete genes in the output of Cufflinks and MAKER, respectively.

**Table 3:** Completeness results done by BUSCO against restricted Arthropoda dataset.

Cufflinks						
	MvM^a^		PvM^b^		Filtered PvM^c^	
**Complete**	308	64.7%	300	63.0%	304	63.9%
*Single copy*	261	54.8%	258	54.2%	264	55.5%
*Duplicated*	47	9.9%	42	8.8%	40	8.4%
**Fragmented**	99	20.8%	102	21.4%	100	21.0%
**Missing**	69	14.5%	74	15.5%	72	15.1%
**476 total BUSCO genes**						
**MAKER**						
	**MvM** ^**a**^		**PvM** ^**b**^		**Filtered PvM** ^**c**^	
**Complete**	382	80.3%	384	80.7%	382	80.3%
*Single copy*	382	80.3%	384	80.7%	382	80.3%
*Duplicated*	0	0%	0	0%	0	0%
**Fragmented**	46	9.7%	44	9.2%	45	9.4%
**Missing**	48	10.1%	48	10.1%	49	10.3%
**476 total BUSCO genes**						

^a^*MvM* refers to male RNA mapped against the male genome assembly. ^b^*PvM* refers to pool RNA mapped against the male genome. ^c^*Filtered PvM* refers to filtered pool RNA mapped against the male genome.

Overall, the mapping of the transcripts to the worker genome resulted in much worst gene models (See Supplementary Table S6). The male and pool transcripts mapped to the worker genome resulted in 24.6% and 23.5% complete genes respectively, as opposed to 64.7% and 63.0% when mapped to the male genome.

**Figure 2:**
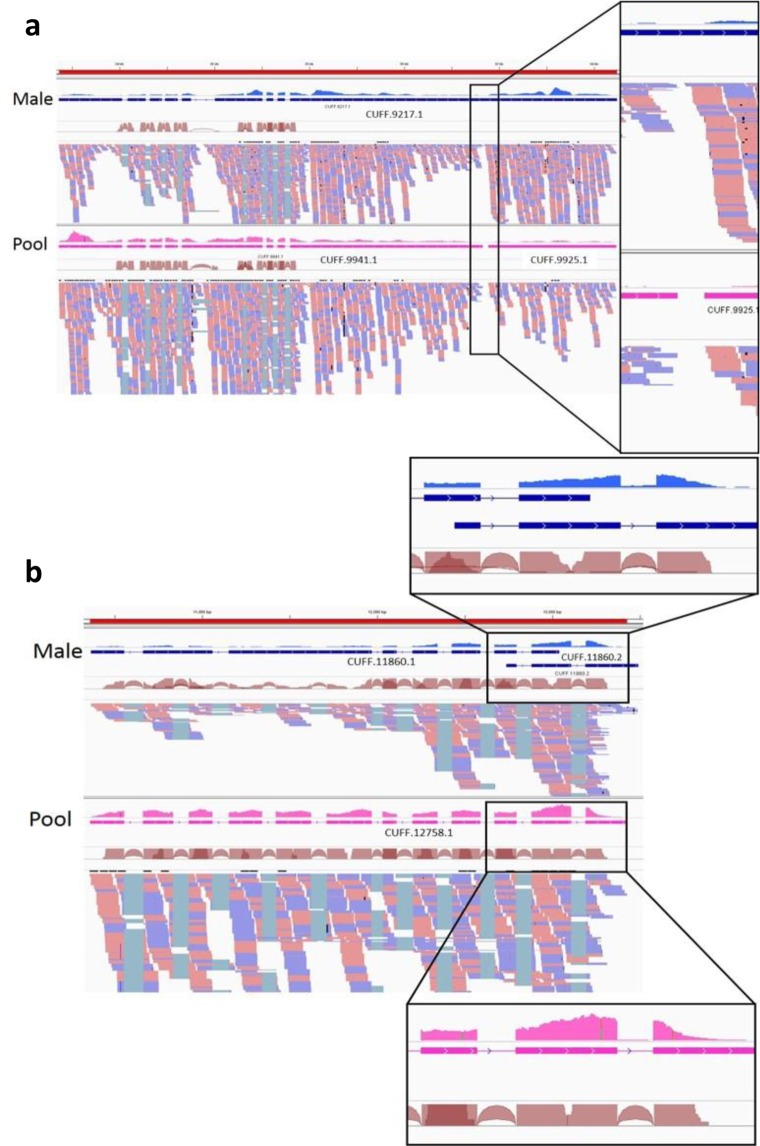
Fragmented vs. complete transcripts in the comparison of the male and pool transcriptomes. (**a**) An example for a gene classified by BUSCO as complete in the male and fragmented in the pool (BUSCO gene EOG09370DXT). The coverage data range is normalized to a range of 0–2500 reads per position. (**b**) An example for a gene classified by BUSCO as complete in the pool and fragmented in the male (BUSCO gene EOG093706PM). The coverage data range is normalized to a range of 0–1000 reads per position.

Of the 476 genes, 238 were complete gene models in both male and pool transcriptomes, and the rest were either fragmented or missing in one or both datasets. Manual inspection and comparison of 80 gene models in the two transcriptomes revealed that the main differences were due to missing exons or genes that were split to two or more separate models. Figure 2a shows an example that BUSCO classified as complete in the male (transcript CUFF.9217) while fragmented in the pool. In the pool sample, the coverage decreases dramatically in the segment between the fragmented transcripts CUFF.9941 and CUFF.9925, leaving a gap of 110 bp with no RNAseq reads mapped, compared to a gap of 22 bp in the male transcriptome in the same position. This may have resulted in Cufflinks failing to recognize them as part of the same gene. Coverage reduction near the edges of gene fragments appear in most genes classified as fragmented by BUSCO. Figure 2b shows a gene that was classified as fragmented in the male and complete in the pool sample. Transcript CUFF.11860.1 and CUFF.11860.2 in the male were not combined by Cufflinks as in the pool transcript CUFF.12758.1.

**Figure 3:**
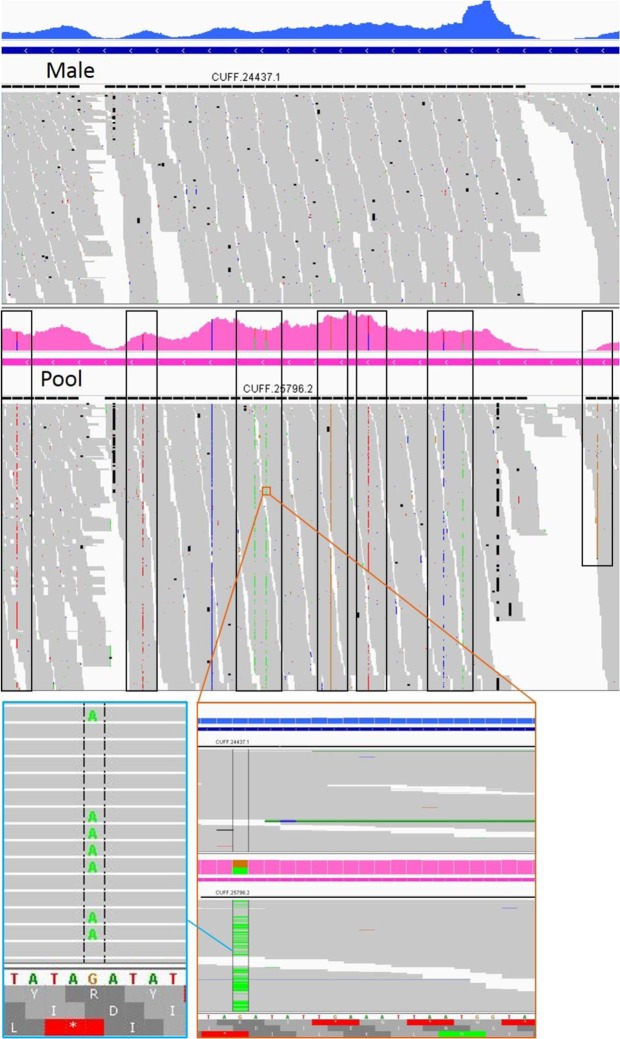
An example of SNPs in the pool transcriptome. The male genome and transcriptome both have a G at this position, while the pool RNAseq reads, have either A or G. The black rectangles highlight multiple additional SNPs.

As expected, many SNPs were observed in the pool and none in the male RNAseq reads, which may be one of the factors contributing to better gene annotation based on the male transcriptome. Any base mismatches in the male sample could be explained by sequencing errors, since they appeared only sporadically in a few of the reads. Figure 3 shows an example of several positions, which show SNPs in the pool sample only. Complexity associated with alternative splice variants may also be contributing to fragmentation of transcripts assembled by Cufflinks.

**Figure 4:**
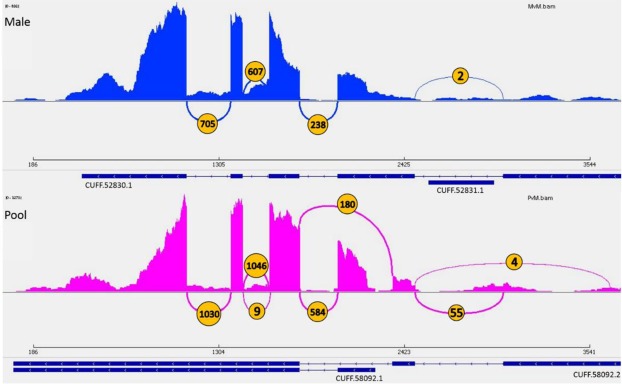
An example of alternative splicing in the pool and male transcriptomes. Visualization of splice junctions using IGV Sashimi plot of male and pool transcripts of same gene. All the splice junctions are of on the negative strand of gene EOG093710JH. Arcs represent splicing events. In orange circles are the number of reads splits across the splice junction. Height of bars between arcs represents exon coverage (reads per position).

Figure 4 shows a as a representative example of alternative splice junctions, as inferred by Cufflinks from the two transcriptomes. The number of splice variants, as indicated by the number of the arcs, was higher in the pool sample than the male.

## Discussion

This study evaluated the utility of haploid samples as the source for both genomic and transcriptomic material in a *de novo* genome sequencing project. These benefits are reflected in multiple aspects of accuracy and completeness of the genomic draft. To date, over 20 hymenopteran genomes were fully sequenced and assembled^29^. A few, such as the leafcutter ant *Acromyrmex echinatior*^8^ and the fire ant *Solenopsis invicta*^9^, used haploid males as their main source for the assembly, alongside a pool of workers for mate pair libraries and RNAseq. Nevertheless, our study is the first to quantitatively assess the quality of assembly when using a haploid male as the source for DNA and the advantage of using RNA and DNA from the same haploid male.

### Genome assembly

Sequence polymorphism has long been recognized as a major challenge for genome assembly of diploid organisms^30^, and even more so for polyploid organisms^7^. One of the most polymorphic diploid organisms that was sequenced is the chordate amphioxus, with 3.7% SNPs and 6.8% polymorphic insertions/deletions^4^. This genome was successfully assembled by combining deep Sanger sequencing with sequencing of a bacterial artificial chromosome (BAC) library. Clearly, such great efforts and investment cannot be made for every non-model organism. Therefore, it is beneficial to make use of a DNA source of lower ploidy level, where possible.

The greatest advantage of the haploid sample we observed was as the source for genomic DNA for the genome assembly. Both SPAdes and SOAPdenovo2 achieved much greater contig and scaffold N50 sizes, by a three to ten-fold factor, for the haploid relative to the diploid sample, without any substantial cost such as more misassemblies. Our results are in line with a previous study by Zhang *et al*.^31^ that used lab-reared double-haploid fish (*Takifugu rubripes*), which are equivalent to the haploid males of Hymenoptera. They compared genome assemblies using double-haploid and wildtype diploid samples, and reported an increase in N50 size by 5.7 and 2.6 fold for contigs and scaffold, respectively. These results support the conclusion that the lack of polymorphism in the haploid DNA sample greatly facilitates assembly of longer contiguous genomic segments, whereas heterozygous sites in the diploid sample confuse the assembler by presenting multiple paths for contig extension.

Furthermore, the size of the diploid SPAdes assembly (759 Mb) is highly overestimated compared to the 220 Mb estimate by flow cytometry. A likely main factor contributing to this bloating of the genome is polymorphic sequences (including SNPs, insertions/deletions, repetitive elements, rearrangements, etc.) that were assembled separately for the two haplotypes of the diploid sample. This interpretation is supported by the high percentage (78%) of duplicated genes found by BUSCO (Table 2). The bloating of the genome and the number of duplicated genes are most likely due to the difficulty for the assembler in dealing with large eukaryotic, diploid genomes. SPAdes was originally designed for small, less repetitive bacterial genomes. An extension of SPAdes, called dipSPAdes, that was designed for dealing with highly polymorphic diploid genomes, might have been a more capable solution^32^.

The lack of longer insert size libraries in this study (i.e. mate-pair sequencing) means the assembler had limited ability for scaffolding longer scaffolds and by that to increase the scaffold N50 size. Nevertheless, our study is a fair comparison of diploid and haploid DNA sources, and is informative regarding the advantage of using a haploid DNA source, at least for the contiging stage of the assembly.

The large number of local misassemblies in both haploid and diploid SOAPdenovo2 assemblies compared to SPAdes can be associate with each assembler’s application of the *de-Bruijn* graph approach. The unique PDBG approach of *k*-bimer adjustment used by SPAdes, helps to avoid misassemlies created by chimeric read pairs^17^. Apparently, the standard *de-Bruijn* approach used by SOAPdenovo2 is more sensitive to this issue.

### Transcripts to genome mapping and gene annotation

Naturally, the pool sample includes genes that are not expressed in the male sample, either because they are specific to certain developmental stages (larvae and pupae) or to the other castes (workers, gynes and queens). When considering genes with similar expression levels in both male and pool transcriptomes and comparing their transcript assembly by Cufflinks (before MAKER annotation), the male RNAseq data resulted in more complete transcripts. Most of the genes classified as fragmented or missing by BUSCO were the result of split transcripts (based on manual inspection). This may be attributed to a drastic coverage drop at a certain point along the transcript, which leads Cufflinks to split the gene to two fragments. The improvement in gene models of both samples after annotation by MAKER can be attributed to MAKER’s use of alignment to homologous proteins from other species. We attribute the higher success of Cufflinks with the male transcriptome to the lack of mismatches between the RNA and DNA sequences. This led to an interesting idea (by an anonymous reviewer): to filter RNAseq reads from the pool that have 100% identity to the genome. The results were intermediate between the results of the male and the unfiltered pool. This approach mimics the sequencing of RNA from the same individual, without the technical difficulty of obtaining RNA and DNA from the same sample, and with the advantage of including transcripts from all life stages and castes.

As described in section 3.2.3, the RNA pool is composed of several castes and developmental stages. This introduces additional complexity to the pool transcriptome relative to the simpler male transcriptome, in terms of both polymorphism and higher diversity of splice isoforms (caste-specific or developmental-stage-specific splicing). In several fragmented genes Cufflinks did not assemble the transcripts correctly or fully even though the raw reads did align well to the genome. Therefore, it may be advisable to avoid the use of a pooled sample. In order to lower the complexity of the pool each sample should be sequenced and assembled separately by Cufflinks. Later all assemblies can be combined using Cuffmerge^22^.

RNA editing is another factor that may add to the complexity of RNAseq data. RNA editing is a post-transcriptional mechanism that modifies some or all of the population of transcripts from a single genomic locus. The most common RNA editing mechanisms is de-amination of adenosine to inosine (A-to-I editing). Inosine is recognized by the ribosome as guanidine, and is also read as such by the polymerase^33,34^. Thus, I is read as a G in RNAseq reads. In ants, 8–23% of overall RNA editing sites are conserved and were suggested as a possible mechanism that contributed to the evolution of sociality^34^. RNA editing levels vary among castes, and thus possibly underlie caste differences in morphology, physiology, and behavior. Therefore, this variation may add further difficulty in the analysis of the pool RNAseq data.

## Conclusion

The ploidy of the source material is an important factor in the design of a *de novo* genome sequencing project. *De novo* assembling a genome using a haploid source (when possible as in the case of Hymenoptera) yields substantially better results in terms of genome contiguity and correct representation of the full gene set without duplications. The use of RNA from the same individual for gene annotation provides minor improvement in the transcripts for the genes that are expressed in that individual. Naturally, RNA sequencing from additional individuals are still needed to obtain a complete gene set including genes that are specific to other life states, sexes and castes. To conclude, hymenopteran genome projects are advised to use the DNA of a haploid individual, however sequencing the RNA of the same individual does not provide a substantial advantage.

## Supplementary information

Supplementary information

## Data Availability

All data generated during this study including genome assembly, transcriptome assembly and raw sequencing data was submitted to NCBI. The raw sequencing data was submitted under BioProject accession PRJNA494690. The genome assembly of the male sample was submitted under accession SJPC00000000.

## References

[1] Church DM (2011). Modernizing reference genome assemblies. PLoS Biol..

[2] Sims D, Sudbery I, Ilott NE, Heger A, Ponting CP (2014). Sequencing depth and coverage: key considerations in genomic analyses. Nat. Rev. Genet..

[3] Simpson JT, Pop M (2015). The Theory and Practice of Genome Sequence Assembly. Annu. Rev. Genomics Hum. Genet..

[4] Putnam NH (2008). The amphioxus genome and the evolution of the chordate karyotype. Nature.

[5] Steinberg, K. M. *et al*. Single haplotype assembly of the human genome from a hydatidiform mole Single haplotype assembly of the human genome from a hydatidiform mole. 2066–2076, 10.1101/gr.180893.114.2066 (2014). 10.1101/gr.180893.114 PMC4248323 25373144

[6] Graf, J.-D. & Kobel, H. R. In *Xenopus laevis: Practical Uses in* Cell *and Molecular Biology* (eds Kay, B. K. & Peng, H. B. B. T.-M. in C. B.) **36**, 19–34 (Academic Press, 1991).

[7] Brenchley R (2012). Analysis of the bread wheat genome using whole-genome shotgun sequencing. Nature.

[8] Nygaard S (2011). The genome of the leaf-cutting ant Acromyrmex echinatior suggests key adaptations to advanced social life and fungus farming. Genome Res..

[9] Wurm Y (2011). The genome of the fire ant Solenopsis invicta. Proc. Natl. Acad. Sci. USA.

[10] Eyer PA, Seltzer R, Hefetz A (2017). Molecular Phylogenetics and Evolution An integrative approach to untangling species delimitation in the Cataglyphis bicolor desert ant complex in Israel. Mol. Phylogenet. Evol..

[11] Brodetzki, T. R. *et al*. Incipient species or social polymorphism? Diversity in the desert ant Cataglyphis. 1–51 (2018).

[12] Timmermans I, Grumiau L, Hefetz A, Aron S (2009). Mating system and population structure in the desert ant Cataglyphis livida. Insectes Soc..

[13] Aron Serge, De Menten Ludivine, Van Bockstaele Dirk (2003). Brood sex ratio determination by flow cytometry in ants. Molecular Ecology Notes.

[14] Andrews, S. & others. FastQC: a quality control tool for high throughput sequence data (2010).

[15] Bolger AM, Lohse M, Usadel B (2014). Trimmomatic: A flexible trimmer for Illumina sequence data. Bioinformatics.

[16] Luo R (2012). SOAPdenovo2: an empirically improved memory-efficient short-read de novo assembler. Gigascience.

[17] Bankevich A (2012). SPAdes: A New Genome Assembly Algorithm and Its Applications to Single-Cell Sequencing. J. Comput. Biol..

[18] Medvedev P, Pham S, Chaisson M, Tesler G, Pevzner P (2011). Paired de Bruijn graphs: A novel approach for incorporating mate pair information into genome assemblers. *Lect. Notes Comput. Sci. (including Subser. Lect. Notes Artif. Intell. Lect. Notes*. Bioinformatics).

[19] Gurevich A, Saveliev V, Vyahhi N, Tesler G (2013). QUAST: Quality assessment tool for genome assemblies. Bioinformatics.

[20] Simão FA, Waterhouse RM, Ioannidis P, Kriventseva EV, Zdobnov EM (2015). BUSCO: user guide. Bioinformatics.

[21] der Auwera GA (2013). From FastQ Data to High-Confidence Variant Calls: The Genome Analysis Toolkit Best Practices Pipeline. Curr. Protoc. Bioinforma..

[22] Trapnell C (2012). Differential gene and transcript expression analysis of RNA-seq experiments with TopHat and Cufflinks. Nat. Protoc..

[23] Langmead B, Salzberg SL (2012). Fast gapped-read alignment with Bowtie 2. Nat. Methods.

[24] Trapnell C, Pachter L, Salzberg SL (2009). TopHat: Discovering splice junctions with RNA-Seq. Bioinformatics.

[25] Kim, D. *et al*. TopHat2: accurate alignment of transcriptomes in the presence of insertions, deletions and gene fusions. 1–13 (2013). 10.1186/gb-2013-14-4-r36 PMC4053844 23618408

[26] Trapnell C (2010). Transcript assembly and quantification by RNA-Seq reveals unannotated transcripts and isoform switching during cell differentiation. Nat Biotech.

[27] Robinson JT (2011). Integrative genomics viewer. Nat. Biotechnol..

[28] Cantarel BL (2008). MAKER: An easy-to-use annotation pipeline designed for emerging model organism genomes. Genome Res..

[29] Elsik CG (2016). Hymenoptera Genome Database: Integrating genome annotations in HymenopteraMine. Nucleic Acids Res..

[30] Vinson J. P. (2005). Assembly of polymorphic genomes: Algorithms and application to Ciona savignyi. Genome Research.

[31] Zhang H (2014). Dramatic improvement in genome assembly achieved using doubled-haploid genomes. Sci. Rep..

[32] Safonova Yana, Bankevich Anton, Pevzner Pavel A. (2014). dipSPAdes: Assembler for Highly Polymorphic Diploid Genomes. Lecture Notes in Computer Science.

[33] Laurencikiene J, Källman AM, Fong N, Bentley DL, Ohman M (2006). RNA editing and alternative splicing: the importance of co-transcriptional coordination. EMBO Rep..

[34] Li Q (2014). Caste-specific RNA editomes in the leaf-cutting ant Acromyrmex echinatior. Nat. Commun..

