# Supplementary material for: A comparative analysis of methods for de novo assembly of hymenopteran genomes using either haploid or diploid samples
Source: Sci Rep. 2019 Apr 24;9:6480. doi: 10.1038/s41598-019-42795-6 (PMC6482151; doi:10.1038/s41598-019-42795-6)
Supplement: Supplementary file 1 — Supplementary information [file 41598_2019_42795_MOESM1_ESM.pdf]

Supplementary material:

A comparative analysis of methods for *de novo* assembly of hymenopteran genomes using either haploid or diploid samples

Tal Yahav, Eyal Privman[#]

Department of Evolutionary and Environmental Biology, Institute of Evolution, University of Haifa, Haifa 3498838, Israel

# Corresponding author: Eyal Privman eprivman@univ.haifa.ac.il

Content:

Supplementary figure S1
Supplementary tables S2 – S6
Supplementary files S7 – S8
Supplementary references

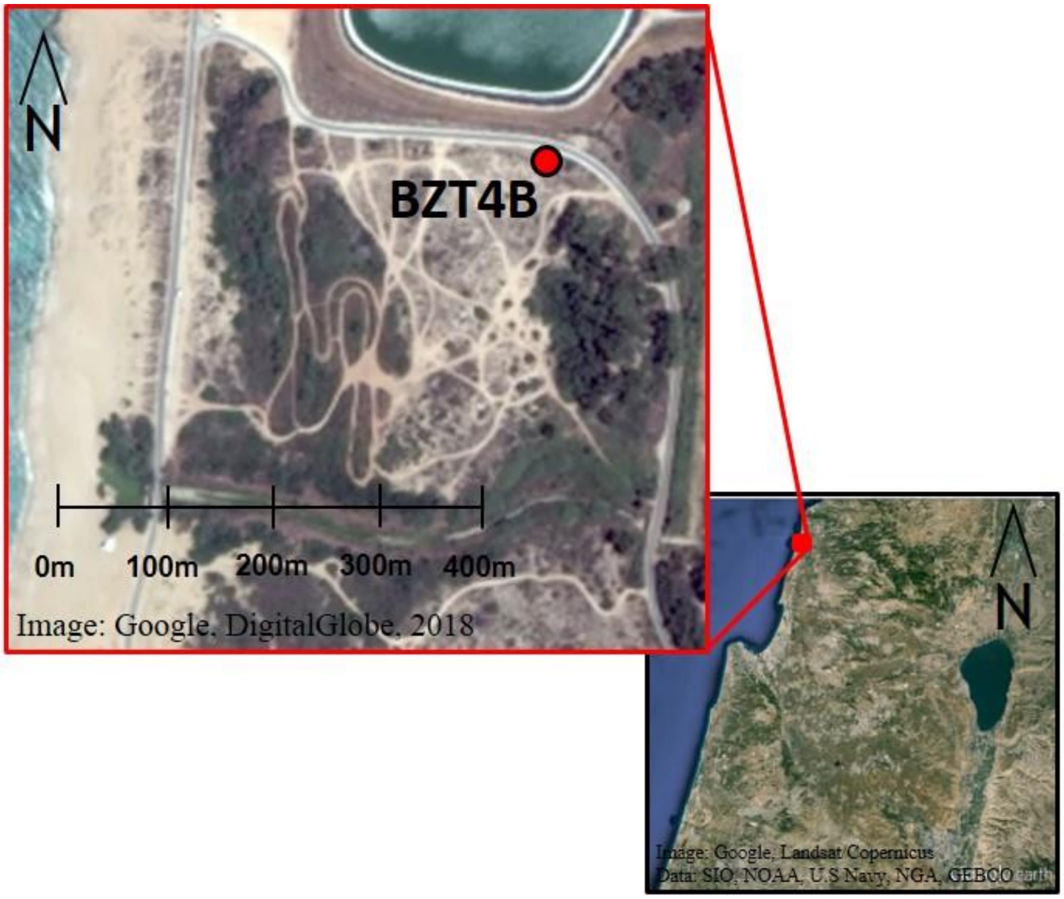

Figure S1: a satellite image of the research area in *Bezet* beach (33°4'40.88"N / 35°6'33.97"E; Google earth). Red dot marks nest BZT4B, from which the male and worker sample for the reference genome were taken.

Table S2: RNA pool composition with quantity and purity measurements using ND200. Total volume of pool sample for sequencing was 60µl. only four samples were diluted (Larva_1, Larva_2, Larva_4, Larve_5). 260/280 ratio < 2 indicate protein residue contamination. 260/230 ratio < 2.2 indicates chemical contamination.

Sample type	Nucleic Acid concentration [ng/µl]	260/280 ratio	260/230 ratio	Total amount of RNA in sample [ng/50µl]	concentration After x10 dilusion [ng/µl]	volume taken after dilusion [µl]
Larva_1 (small size)	1052.3	2.13	2.24	52615	105.23	5
Larva_2 (small size)	1790	2.13	2.09	89500	179	3
Larva_3 (medium size)	661.6	2.08	2.01	33080	N/A	1
Larva_4 (medium size)	1243.2	2.11	1.77	62160	124.32	5
Larva_5 (large size)	1356.3	2.12	1.97	67815	135.63	5
Pupa_1 (small size)	83.8	2.08	0.92	4190	N/A	7
Pupa_2 (small size)	102.7	2.11	0.94	5135	N/A	5
Pupa_3 (large size)	215.1	2.11	1.15	10755	N/A	5
Gyne	57.2	2.06	0.89	2860	N/A	20
Male	146.5	2.2	1.85	7325	N/A	10
Worker	105.7	2.02	1.1	5285	N/A	10
Total RNA_POOL-A sample	106.6	2.03	1.08			60
Total RNA_POOL-B sample	102	2.01	1.11			60

Table S3: DNA and RNA ND2000 measurements results for the male candidate samples and Worker DNA measurements.

Sample type	Nucleic Acid concentration [ng/µl]	260/280 ratio	260/230 ratio	Total amount of DNA/RNA in sample [ng/40µl]
DNA				
Male_BZT4B	121.824	2.229	0.649	4872.96
Male_BZT4C	45.652	2.227	0.735	1826.08
Male_BZT7	44.861	2.186	0.899	1794.44
Worker_BZT4B	278.8	2.12	1.9	8364
RNA				
BZT4B_M1	660.113	2.274	1.512	26404.52
BZT4C_M2	1038.168	2.26	2.247	41526.72
BZT7_M2	1065.382	2.277	1.518	42615.28

Table S4: Species homolog proteins used for MAKER annotation

Species name	Databae
Acromyrmex echinatior	http://www.antgenomes.org
Atta cephalotes	http://www.antgenomes.org
Harpegnathos saltator	http://www.antgenomes.org
Lasius niger	http://www.antgenomes.org
Linepithema humile	http://www.antgenomes.org
Pogonomyrmex barbatus	http://www.antgenomes.org
Drosophila melanogaster	https://www.ncbi.nlm.nih.gov/
Apis mellifera	https://www.ncbi.nlm.nih.gov/
Polistes dominula	https://www.ncbi.nlm.nih.gov/

Table S5: Microsatellites used for ploidy test of the male samples. *C. niger* (Cn02 and Cn04), C. *hyspanica* **(**Ch08**)**[S1] and *C. cursor* (Cc54)[S2].

Locus	Dye	Expected size	Homozygosity*	Primer sequence
**Cn02**	PET	115 bp	0.682	Forward 5'=>3' GAGGCCCCTGAAAAGAAGAT<br>Reverse 5'=>3' TTCTATCTCTGCCGGCTTCT
**Cn04**	VIC	95 bp	0.251	Forward 5'=>3' GGAAACTCGTGCGAAAACTC<br>Reverse 5'=>3' GAGCTCAGTGTGCATTCAACAT
**Ch08**	NED	135 bp	0.289	Forward 5'=>3' GCTGATAATCGCGTCTGGAT<br>Reverse 5'=>3' CGACGTAAAGAGGAACGTGA
**Cc54**	FAM	210 bp	0.553	Forward 5'=>3' GAATTTGAATGGCTGATTGC<br>Reverse 5'=>3' ATGGTCGTTTGGCATAAAGG

***** Proportion of homozygous out of a total of 708 samples in the *Bezet* site.

Table S6: Completeness results by BUSCO against restricted Arthropoda dataset

Cufflinks				
	MvW[a]		PvW[b]	
**Complete**	117	24.6%	112	23.5%
Single copy	97	20.4%	83	17.4%
Duplicated	20	4.2%	29	6.1%
**Fragmented**	173	36.3%	184	38.7%
**Missing**	186	39.1%	180	37.8%
**476 total BUSCO genes**				

(a) *MvW* refers to male RNA mapped against the worker genome assembly. (b) *PvW* refers to pool RNA mapped against the worker genome.

File S7: All Prep Mini DNA and RNA modified protocol for *Cataglyphis* ants

Amount of starting material Volume of Buffer RLT
20–30 mg 600 µl (an average *cataglyphis* worker is ~20 mg)

Tissue disruption:

1. Disrupt the tissue and homogenize the lysate in RLT.
*** add 10 µl of β-ME to 1ml of RLT buffer before use.

Disruption and homogenization using the Tissue Lysser >>> 3 metal beads + shredded glass powder + glass beads (SIGMA) inside 1.5-2 ml tube. Snap frozen tissue is put inside tube and in liquid nitrogen before disruption (can be repeated).

Tissue Lysser >>> 30 Hz for cycles of 20 sec (or 50 sec) **dry cycles** + **wet cycles** with addition of 100ul of RLT+ β-ME.

After Tissue Lysser add 700 µl of RLT and incubate in 65°C for 30-60 min.

2. Centrifuge >>> lysate for 3 min, max speed.
Carefully remove the supernatant by pipetting, and transfer it to the AllPrep DNA spin column placed in a 2 ml collection tube.

Centrifuge >>> 30 sec, max speed (can use ~ 10,000 g)

3. Place the AllPrep DNA spin column in a new 2 ml Eppendorf tube and store at 4°C for later DNA purification. **Do not freeze the column**.

***Use the flow-through for RNA purification.

Total RNA purification:

4. Add 1 volume (~700 µl) of 100% frozen ethanol (instead of 70%) to the flow through, and mix well by pipetting. **Do not centrifuge.** Incubate in -20°C for 10-15min.

5. Transfer up to 700 µl of the sample, including any precipitate that may have formed, to an RNeasy spin column placed in a 2 ml Collection tube.

Centrifuge >>> 30 sec, max speed (Discard the flow through).

*** If the sample volume exceeds 700 µl, centrifuge successive aliquots in the same RNeasy spin column. Discard the flow-through after each centrifugation.

DNase Treatment (optional):

E1. Add 350 µl Buffer RW1 to the RNeasy spin column, and centrifuge for 15 s at ≥8000 x *g* (≥10,000 rpm) to wash the spin column membrane. Discard the flow-through.*

Reuse the collection tube in step E4.

E2. Add 10 µl DNase I stock solution (see above) to 70 µl Buffer RDD. Mix by gently inverting the tube, and centrifuge briefly to collect residual liquid from the sides of the tube.
Buffer RDD is supplied with the RNase-Free DNase Set.
Note: DNase I is especially sensitive to physical denaturation. Mixing should only be carried out by gently inverting the tube. Do not vortex.

E3. Add the DNase I incubation mix (80 µl) directly to the RNeasy spin column membrane, and incubate at room temperature (20–30°C) for 15 min.

***Note:** Be sure to add the DNase I incubation mix directly to the RNeasy spin column membrane. DNase digestion will be incomplete if part of the mix sticks to the walls or the O-ring of the spin column.

E4. Add 350 µl Buffer RW1 to the RNeasy spin column, and centrifuge for 15 s at ≥8000 x *g* (≥10,000 rpm). Discard the flow-through.*
Continue with step 9 of the protocol on page 26 (i.e., the first wash with Buffer RPE).
Reuse the collection tube in step 9.

Optional:
6. Add 700 µl Buffer RW1 to the RNeasy spin column to wash the spin column membrane.

Centrifuge >>> 30 sec, max speed (Discard the flow-through).

7. Add 450 µl RPE to the RNeasy spin column (can do 2-3 washes)
Reuse the collection tube in step 10.

8. Add 450 µl RPE to the RNeasy spin column.

Centrifuge >>> 2 min, max speed (Discard the flow-through).

9. Place the RNeasy spin column in a new 2 ml collection tube (discard the old collection tube with the flow through).

Centrifuge >>> 1 min, max speed.

10. Place the RNeasy spin column in a new 1.5-2 ml collection tube Add 40 µl RNase-free water directly to the spin column membrane. Incubate for 10-15 min in RT.

Centrifuge >>> 1 min, max speed.

Genomic DNA purification:

11. Add 500 µl Buffer AW1 to the AllPrep DNA spin column from step 5.

Centrifuge >>> 30 sec, max speed. (Discard the flow-through).

Note: Buffer AW1 is supplied as a concentrate. Ensure that ethanol is added to Buffer AW1 before use.

12. Add 500 µl Buffer AW2 to the column.
Centrifuge >>> 2 min, max speed. (Discard the flow-through).

13. Place the AllPrep DNA spin column in a new 1.5 ml collection tube
Add 50 µl Buffer EB (preferably use 2mM tris heated to 55°C) directly to the spin column membrane and close the lid. Incubate 10-15 min at RT.

Centrifuge >>> 1min, max speed.

File S8: List of commands used in the various pipelines in the genome and transcriptome assembly as well as quality assessment.

```
'java -jar trimmomatic-0.36.jar PE -phred33 ILLUMINACLIP:TruSeq3-PE.fa:2:30:10 LEADING:3 TRAILING:3 SLIDINGWINDOW:4:15 MINLEN:80'
```

```
'java -jar trimmomatic-0.36.jar PE -phred33 ILLUMINACLIP:TruSeq3-PE.fa:2:30:10 LEADING:3 TRAILING:3 SLIDINGWINDOW:4:15 MINLEN:60'
```

```
'spades.py --pe1-1 < 300bp R1.fastq > --pe1-2 < 300bp R2.fastq > --pe2-1 < 550bp R1.fastq > --pe2-2 < 550bp R2.fastq > -k 13,23,33,43,53,63,73,83,93,103,113,123 -t 20 -o <output folder>'
```

```
'SOAPdenovo-127mer all -s cdru_W4B.config -K 115 -R -p 20 -o cdru_W4B 1>cdru_W4B.log 2>cdru_W4B.err'
```

```
'python3 BUSCO_v1.22.py -o dru_M4B_4_3_2017_SOAP -in /data/home/ASSEMBLIES/SOAPdenovo2/cdru_M4B_28_2_2017/cdru_M4B.scafSeq  -l /data/home/SOFTWARES/BUSCO_v1.22/arthropoda -m genome -c 20'
```

```
'python quast.py -e -t 20 -o /data/home/ASSEMBLIES/QUAST/cdru_W4B_spades_4_3_2017 /data/home/ASSEMBLIES/SPades/W4B_K13-123_28_2_2017_B/scaffolds.fasta /data/home /ASSEMBLIES/SPades/W4B_K13-123_28_2_2017_B/contigs.fasta -R /data/home /ANTS_RAW_DATA/ANTS_ASSEMBLIES/Chis1_v1.0.sorted.fa'
```

```
'bowtie2-build --threads 20 -f /data/home/ASSEMBLIES/SPades/W4B_K13-123_28_2_2017_B/scaffolds.fasta cdru_W4B'
```

```
'gffread transcripts.gff3 -g /data/home/C_DRUSUS_TAL/ASSEMBLIES/DNA/SPades/M4B_K13-123_28_2_2017_B/scaffolds.fasta -y transcripts_2.fasta -M'
```

```
'sed -e 1~16d;2~16d;3~16d;4~16d'
```

```
'tophat -p 20 M4B_K13-123_28_2_2017_B_scaffolds /data/home/C_DRUSUS_TAL/7_RNA_M4B_PE125_IS500/RAW_DATA/RNA_M4B_Pst_R1.fastq /data/home/C_DRUSUS_TAL/7_RNA_M4B_PE125_IS500/RAW_DATA/RNA_M4B_Pst_R2.fastq'
```

```
'cufflinks --no-update-check -p 20 /data/home/ASSEMBLIES/RNA/bowtie2_spades_12_8_2017/M4B_Vs_M4B_asm/tophat_out/accepted_hits.bam'
```

Supplementary references

S1. Darras, H., Leniaud, L. & Aron, S. Large-scale distribution of hybridogenetic lineages in a Spanish desert ant. *Proc. R. Soc. B* **281,** 20132396 (2014).

S2. Pearcy, M., Clémencet, J., Chameron, S., Aron, S. & Doums, C. Characterization of nuclear DNA microsatellite markers in the ant Cataglyphis cursor. *Mol. Ecol. Notes* **4,** 642–644 (2004).
